# Effects of Transgenerational Plasticity on Morphological and Physiological Properties of Stoloniferous Herb *Centella asiatica* Subjected to High/Low Light

**DOI:** 10.3389/fpls.2018.01640

**Published:** 2018-11-14

**Authors:** Kenian Li, Jinsong Chen, Qing Wei, Qian Li, Ningfei Lei

**Affiliations:** ^1^College of Life Science, Sichuan Normal University, Chengdu, China; ^2^College of Environment, Chengdu University of Technology, Chengdu, China

**Keywords:** maternal effects, potential maximum net photosynthetic rate (*P_max_*), leaf nitrogen content, internode length of stolon, leaf area

## Abstract

Environmentally induced transgenerational plasticity can increase success of progeny and thereby be adaptive if progeny experiences the similarly parental environment. The ecological and evolutionary significance of transgenerational plasticity in plant has been studied mainly in the context of sexual generations. A pot experiment using the stoloniferous herb *Centella asiatica* was conducted to investigate the effects of high/low light treatment experienced by parental ramets (F_0_ generation) on morphological and physiological properties of offspring ramets (F_2_ generation) as well as growth performance. Light environment experienced by parental ramets (F_0_ generation) significantly influenced petiole length, specific petiole length, internode length of stolon, leaf area, specific leaf area (SLA), leaf nitrogen and chlorophyll contents, potential maximum net photosynthetic rate (*P_max_*) in offspring ramets subjected to parental or non-parental environments even after they were detached from the parental ramets. Potential maximum net photosynthetic rate (*P_max_*) of offspring ramets (F_2_ generation) from parental ramets (F_0_ generation) subjected to low light treatment was significantly greater than that of offspring ramets (F_2_ generation) from parental ramets (F_0_ generation) subjected to high light treatment. Potential maximum net photosynthetic rate (*P_max_*) of offspring ramets (F_2_ generation) subjected to parental light environment was greater than that of offspring ramets (F_2_ generation) subjected to non-parental light environment. The greatest biomass accumulation and total stolon length were observed in offspring ramets (F_2_ generation) subjected to low light treatment as parental ramets (F_0_ generation) experienced. When parental ramets (F_0_ generation) were subjected to low light treatment, biomass accumulation and total stolon length of offspring ramets (F_2_ generation) experiencing parental light environment were significantly greater than those of offspring ramets (F_2_ generation) experiencing non-parental light environment. Opposite pattern was observed in offspring ramets (F_2_ generation) from parental ramets subjected to high light treatment. Our work provides evidence that transgenerational plasticity through both morphological and physiological flexibility was triggered across vegetative generations for stoloniferous herb *C. asiatica* subjected to high/low light treatment. The transgenerational plasticity can allow offspring ramets to present adaptive phenotype early without lag time in response to the current environment. Thus, it is very important for clonal plants in adapting temporally and spatially heterogeneous habitats.

## Introduction

The environmental cues experienced by parents, may influence the phenotype of their progeny. This phenomenon is termed as transgenerational plasticity (Dyer et al., 2010; Fenesi et al., 2014). Transgenerational plasticity elicits phenotypic adjustments to environmental conditions experienced by sexually produced progeny. For example, soil nutrient conditions encountered by parent, affects size and germination of progeny in *Senecio* sp (Aarssen and Burton, 1990); the competitive ability in *Plantago major* and *P. rugelii* is related to the environmental conditions experienced by both parent and progeny (Miao et al., 1991). The parental light environment influences the life history schedule of progeny in *Campanulastrum americanum* (Galloway and Etterson, 2007). The defensive resistance of progeny is induced by herbivory in the parental generation of *Raphanus raphanistrum* (Agrawal, 2002). Transgenerational plasticity can be mediated by altered DNA methylation (Rossiter, 1996; Douhovnikoff and Dodd, 2015) or seed quality (Roach and Wulff, 1987). Thereby, transgenerational plasticity may be potentially important for evolutionary dynamics of plant population (Riska, 1989; Räsänen and Kruuk, 2007).

Transgenerational plasticity may be adaptive in progeny grown under the same environmental conditions as experienced by parent (Galloway, 2005; Galloway and Etterson, 2007; Chen et al., 2014; Latzel et al., 2014). For the monocarpic herb *C. americanum*, fitness of progeny grown under a parental light environment is significantly greater than that of progeny grown under a non-parental light environment (Galloway and Etterson, 2007). Similarly, nutrient conditions experienced by parent, significantly affects biomass and carbon storage of progeny in *Plantago lanceolata* (Latzel et al., 2014). In addition, transgenerational plasticity may be more important for plant grown under limited resource conditions (such as in soil with low water level and nutrient or in shaded habitat) than one grown under ample resource conditions (Sultan, 1996). However, the ecological and evolutionary significance of transgenerational plasticity mainly focus on studies across sexual generations.

Clonal plant can reproduce a large number of interconnected, potentially independent and genetically identical offspring ramets. Transgenerational plasticity may influence phenotype of offspring ramets (Dong et al., 2017; González et al., 2017). For stoloniferous herb *Trifolium repens*, greater compensatory growth was observed in offspring ramets propagated from parental ramets subjected to repeated application of jasmonic acid compared to ones from parental ramets subjected to the same volume distilled water without application of jasmonic acid (González et al., 2017); similar pattern was still observed in offspring ramets of *Alternanthera philoxeroides* propagated from populations suffering from long-time herbivory disturbance (Lu and Ding, 2012). As an alternative to the slower mechanisms of adaptation through natural selection, transgenerational plasticity may confer ecological advantages to clonal plants against the challenges of current and future rapid environmental changes (Verhoeven and Preite, 2014; Douhovnikoff and Dodd, 2015; Dong et al., 2017).

A greenhouse experiment was conducted to explicitly investigate effects of transgenerational plasticity across vegetative generations on morphological and physiological properties of stoloniferous herb *Centella asiatica* subjected to high/low light treatment. Our first hypothesis is that effects of transgenerational plasticity on morphological and physiological properties persist across vegetative generations. Light may be an important resource for growth, development and reproduction of plants (Madsen and Sand-Jensen, 1994; Wagner et al., 2005; Glover et al., 2015). Morphological plasticity is an adaptive strategy of clonal plants to heterogeneous light conditions. For example, internode extension of stolon and petiole elongation may allow clonal ramets to escape from low light patches and lift leaf blades to higher light zones (Oborny, 1994). The ramets subjected to low light condition can intercept more light by enlarging leaf area (Dong, 1995). So, flexible responses in the internode length of stolon, petiole length and leaf area are crucial for clonal plant in capturing light (Hutchings and Kroon, 1994). As a component of chlorophyll, leaf N content is positively correlated with photosynthetic capacity in plant (Feng et al., 2007; Chen et al., 2015). We predicted that high/low light treatment experienced by parental ramets significantly influenced internode length of stolon, specific internode length of stolon, petiole length, specific petiole length, leaf area, specific leaf area (SLA), leaf nitrogen and chlorophyll contents, potential maximum net photosynthetic rate(*P_max_*) in offspring ramets subjected to parental or non-parental light environments.

Our second hypothesis is that effects of transgenerational plasticity on growth performance are context-dependent. Then, we predicted that biomass accumulation and total stolon length of offspring ramets experiencing parental light environment significantly increased than those of offspring ramets experiencing non-parental light environment. Our third hypothesis is that offspring ramets reproduced from parental ramets subjected to low resource level environment should be favored in parental or non-parental environments. So, we predicted that whether in parental or non-parental light environment, biomass accumulation and total stolon length of offspring ramets from parental ramets subjected to low light treatment significantly increased than those of offspring ramets from parental ramets subjected to high light treatment.

## Materials and Methods

### Plant Material

*Centella asiatica* (Umbelliferae) is a stoloniferous perennial herb, which is generally distributed in ditches, margins of ponds, lawns and roadsides. Each ramet is composed of two zygomorphic leaves with slender petiole. The axillary bud on the vertical stem may grow out and form stolon (Chinese Academy of Sciences, 2004). The stolon usually take roots when in contact with moist substratum, forming a network of stolon above the ground.

Eight original plants of *Centella asiatica* were collected in Chengdu, Sichuan Province, China (30°05′ 31°26′N; 102°54′ 104°53′E) (Table 1). The original plants were at least 1 km away each other. They may or may not differ in genotype.

**Table 1:** Basic information on original plants of *Centella asiatica* in the experiment.

No	Location	Habitat type	Dominant species	Community	Size of original plant	
			of community	transmittance		
					Height	Leaf area
					(cm)	(cm^2^)
1	30°56′N;104°18′E	lawn	*Dichondra repens*	98%	4.2	9.5
2	30°57′N; 104°19′E	lawn	*Cynodon dactylon*	96%	4.5	9.8
3	30°59′N; 104°18′E	lawn	*Centella asiatica*	98%	4.3	9.7
4	30°57′N; 104°18′E	roadsides	*Trifolium repens*	97%	4.4	9.7
5	30°59′N; 104°19′E	roadsides	*Poa annua*	95%	4.7	10.0
6	30°57′N; 104°17′E	roadsides	*Buchloe dactyloides*	96%	4.6	9.9
7	30°99′N; 103°54′E	ditches	*Poa annua + Zoysia japonica*	92%	4.8	10.2
8	30°92′N; 103°56′E	ditches	*Buchloe dactyloides + Cynodon dactylon*	91%	4.9	10.1

In April 2016, they were cultivated in a greenhouse, located in Sichuan Normal University. All pots were filled with substrate (3:1 mixture of humus soil and sand). During the experiment, fertilizer (20% N, 20% P, 20% K; The Scotts Company, United States) was applied to each pot once per week. Tap water was supplied to keep the substrate moist. After 4 months, offspring ramets of each original plant formed a “ramet bank” (EI-Keblawy and Bhatt, 2015).

### Experimental Design

*F_0_ generation* August 2016, two parental ramets with similar size from each “ramet bank” were grown into plastic pots (42 cm × 34 cm × 11 cm) respectively. We standardized size of the ramets by removing extra leaves and cutting the roots (Wang et al., 2013; Dong et al., 2017). One ramet was subjected to high light treatment (full light) and the other was subjected to low light treatment (50% full light). All reproduced ramets in each pot were named as F_0_ generation during 10 weeks period.

*F_1_ generation* Two ramets with similar size were chosen from each F_0_ generation and grown in new pots, respectively. One ramet was subjected to high light treatment and the other was subjected to low light treatment. All reproduced ramets in each pot were named as F_1_ generation during another 10 weeks period.

**FIGURE 1:**
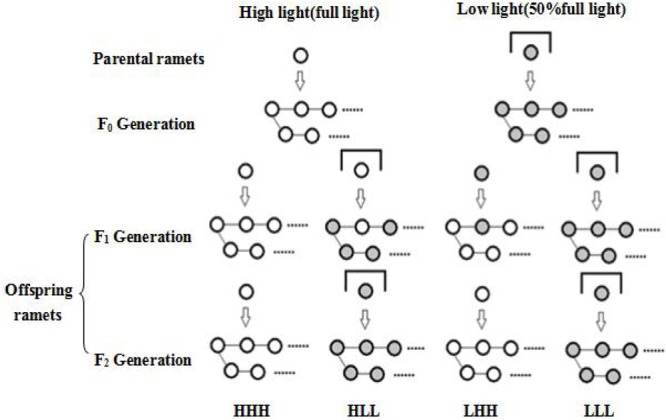
Schematic diagram of the experimental design. The experiment is across three vegetative generations. Four treatments were included for F_2_ generation: F_0_ generation high light + F_1_ generation high light + F_2_ generation high light (HHH); F_0_ generation high light + F_1_generation low light + F_2_ generation low light (HLL); F_0_ generation low light + F_1_ generation high light + F_2_ generation high light (LHH); F_0_ generation low light + F_1_ generation low light + F_2_ generation low light (LLL).

*F_2_ generation* One ramet was chosen from each F_1_ generation and grown in a new pot. The ramet was subjected to light treatment as its F_1_ generation experienced. All reproduced ramets in each pot were named as F_2_ generation during another 10 weeks period. Four treatments were included for F_2_ generation: F_0_ generation high light + F_1_ generation high light + F_2_ generation high light (HHH); F_0_ generation high light + F_1_generation low light + F_2_ generation low light (HLL); F_0_ generation low light + F_1_ generation high light + F_2_ generation high light (LHH); F_0_ generation low light + F_1_ generation low light + F_2_ generation low light (LLL) (Figure 1). There were eight replicates per treatment. The pots were re-randomized to avoid potential effects of environmental heterogeneity. Offspring ramets from each original plant underwent all treatments.

### Morphological Properties

After harvesting, offspring ramets (F_2_ generation) were separated into root, leaf, petiole and stolon. Internode length of stolon and petiole length were measured by ruler. Specific internode length of stolon (internode length of stolon / dry weight) and specific petiole length (petiole length/ dry weight) were counted after drying to constant weight. Leaf area was measured according to the method described by Dong et al. (2015). Specific leaf area (SLA) was counted as follows:

$$\begin{array}{c} Specific leaf area(\mathrm{SLA})=\frac{leaf area}{\mathrm{leaf}\;\mathrm{dry}\;\mathrm{weight}} \end{array}$$

### Photosynthetic Properties

A portable photosynthesis system GFS-3000 (Heinz Walz GmbH, Effeltrich, Germany) was used for measurement of photosynthesis during the last week of growth. Eight offspring ramets (F_2_ generation) with similar size were chosen from each treatment. A fully expanded and mature leaf from each ramet was selected for photosynthetic measurement.

Under a CO_2_ pressure of 400 μmolmol^-1^, a light–response curve [net photosynthesis rate (*P_n_*)–photosynthetic photon flux density (*PPFD*) curve] was generated according to the method described by Chen et al. (2015). The *P_max_* was calculated according to the *P_n_–PPFD* curves which were fitted with a non-rectangular hyperbola model using the plotting software Origin (Origin Lab, United States) (Gomes et al., 2006; Sorrell et al., 2012):

Pn = ϕPPFD+Pmax−(ϕPPFD+Pmax)−4ϕ θPmaxPPFD2θ−Rd

Where *∅* was the apparent quantum efficiency, *𝜃* was the convexity of the curve and *R_d_* was the dark respiration rate.

### Leaf Properties

The leaf for measurement of photosynthetic parameters was then finely ground to determine the nitrogen content with an elemental analyser (vario MACRO CUBE, Elementar Analysensysteme, Hanau, Germany). At the same time, the other zygomorphic leaf originating from the each ramet was selected to measure the absolute chlorophyll content using the dimethylsulphoxide (DMSO) chlorophyll extraction technique (Richardson et al., 2002).

### Growth Performance

After harvesting, dry weights of stolon, petiole, root and leaf were recorded after oven drying at 60°C until constant weight was obtained.

### Statistical Analysis

Prior to analysis, a square root transformation was used for total stolon length and a logarithmic transformation applied to petiole length. Two-way ANOVA was used to investigate the effects of light treatment experienced by F_0_ *generation* (F_0_), light treatment experienced by F_2_ *generation* (F_2_) and their interaction (F_0_ × F_2_) on morphological, leaf and photosynthetic properties of offspring ramets (F_2_ generation) as well as growth performance. Tukey HSD *post hoc* test was empolyed to compare difference among different treatments experienced by F_2_ generation. All analyses were conducted with SPSS 20.0 software (SPSS, Chicago, IL, United States).

## Results

### Morphological Properties

**FIGURE 2:**
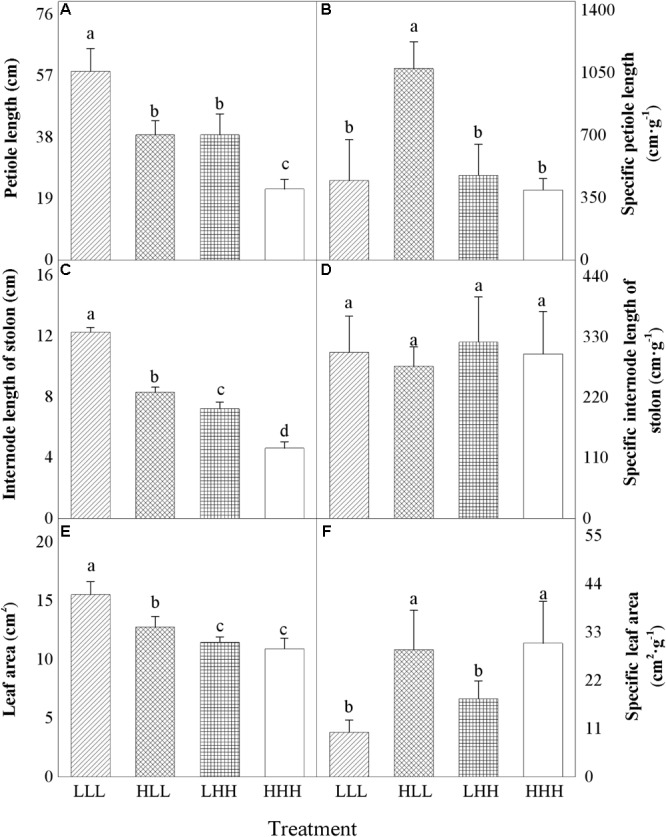
Effects of transgenerational plasticity on morphological properties of offspring ranets (F_2_ generations). The same lower case letters are not significantly different at the *P* = 0.05 level. Values are means ± s.e. (standard errors), *n* = 8. HHH: F_0_ generation high light+ F_1_ generation high light+ F_2_ generation high light; HLL: F_0_ generation high light+ F_1_ generation low light+ F_2_ generation low light; LHH: F_0_ generation low light+ F_1_ generation high light+ F_2_ generation high light; LLL: F_0_ generation low light+ F_1_ generation low light+ F_2_ generation low light.

Specific petiole length and internode length of stolon of offspring ramets (F_2_ generation) were significantly affected by light treatment experienced by F_0_ generation (F_0_), light treatment experienced by F_2_ generation (F_2_) and their interaction (F_0_ × F_2_) (Table 2). Petiole length and leaf area of offspring ramets (F_2_ generation) were significantly affected by light treatment experienced by F_0_ generation (F_0_) and light treatment experienced by F_2_ generation (F_2_) (Table 2). However, specific leaf area (SLA) was significantly affected by light treatment experienced by F_0_ generation (F_0_) (Table 2). We did not detect significant effects of the different treatments on specific internode length of stolon of offspring ramets (F_2_ generation) (Table 2 and Figure 2D).

**Table 2:** Two-way ANOVA results for effects of light treatment experienced by F_0_ generation (F_0_), light treatment experienced by F_2_ generation (F_2_) and their interaction on the morphological, leaf and photosynthetic properties of of offspring ramets (F_2_ generation) as well as growth performance.

Source	df	Morphological properties						Leaf properties				Photosynthetic property	Growth performance	
		Petiole length	Specific petiole length	Internode length of stolon	Specific internode length of stolon	Leaf area	Specific leaf area (SLA)	Area-based leaf chlorophyll content (*ACC_a_*)	Mass-based leaf chlorophyll content (*ACC_m_*)	Leaf nitrogen content per unit mass(*N_A_*)	Leaf nitrogen content per unit area(*N_M_*)	Potential maximum net photosynthetic rate(*P_max_*)	Biomass accumulation	Total stolon length
F_2_	1	40.71^∗∗∗^	7.79^∗^	410.16^∗∗∗^	0.18^ns^	135.80^∗∗^	2.41^ns^	28.87^∗∗^	52.671^∗∗∗^	11.98^∗∗^	9.89^∗^	0.008^ns^	6.25^∗^	13.96^∗∗^
F_0_	1	31.09^∗∗^	5.46^∗^	231.13^∗∗∗^	0.25^ns^	13.94^∗∗^	30.68^∗∗^	22.09^∗∗^	37.44^∗∗∗^	27.82^∗∗^	0.71^ns^	38.18^∗∗∗^	8.05^∗^	18.27^∗∗^
F_0_ × F_2_	1	0.31^ns^	9.23^∗^	9.74^∗^	0.001^ns^	3.62^ns^	0.33^ns^	1.77^ns^	5.40^∗^	6.83^∗^	0.05^ns^	12.32^∗∗^	0.016^ns^	0.03^ns^
Error	28													

*^∗∗∗^*P* < 0.001, ^∗∗^*P* < 0.01, ^∗^*P* < 0.05, ns, non-significant, *P* > 0.05.*

When parental ramets were subject to low light treatment, petiole length and internode length of stolon of offspring ramets (F_2_ generation) experiencing parental light environment significantly increased than those of offspring ramets (F_2_ generation) experiencing non-parental light environment (Figures 2A,C). However, opposite pattern was observed in petiole length and internode length of stolon of offspring ramets (F_2_ generation) from parental ramets subjected to high light treatment (Figures 2A,C). When parental ramets were subject to high light treatment, specific petiole length of offspring ramets (F_2_ generation) experiencing low light environment significantly increased (Figure 2B). Leaf area of offspring ramets (F_2_ generation) experiencing low light treatment significantly increased than that of offspring ramets (F_2_ generation) experiencing high light treatment (Figure 2E). When parental ramets were subject to low light treatment, leaf area of offspring ramets (F_2_ generation) experiencing parental light environment significantly increased than that of offspring ramets (F_2_ generation) experiencing non-parental light environment (Figure 2E). Compared to offspring ramets from parental ramets subjected to high light treatment, specific leaf area (SLA) of offspring ramets (F_2_ generation) from parental ramets subjected to low light treatment significantly decreased (Figure 2F).

### Leaf Properties

**FIGURE 3:**
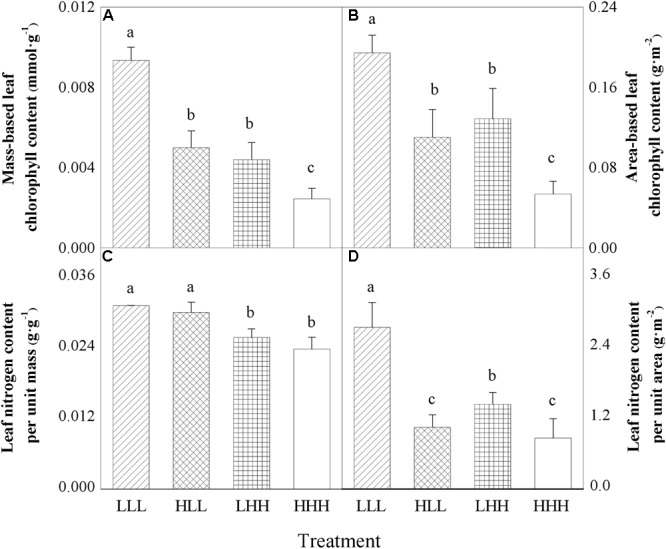
Effects of transgenerational plasticity on leaf properties of offspring ramets (F_2_ generation). The same lower case letters are not significantly different at the *P* = 0.05 level. Values are means ± s.e. (standard errors), *n* = 8. HHH: F_0_ generation high light+ F_1_ generation high light+ F_2_ generation high light; HLL: F_0_ generation high light+ F_1_ generation low light+ F_2_ generation low light; LHH: F_0_ generation low light+ F_1_ generation high light+ F_2_ generation high light; LLL: F_0_ generation low light+ F1 generation low light+ F_2_ generation low light.

Area-based leaf chlorophyll content (*ACC_a_*) of offspring ramets (F_2_ generation) was significantly affected by light treatment experienced by F_0_ generation and light treatment experienced by F_2_ generation (Table 2). Mass-based leaf chlorophyll content (*ACC_m_*) of offspring ramets (F_2_ generation) was significantly affected by light treatment experienced by F_0_ generation, light treatment experienced by F_2_ generation and their interaction (F_0_ × F_2_) (Table 2). When parental ramets (F_0_ generation) were subjected to low light treatment, area-based leaf chlorophyll content (*ACC_a_*) and mass-based leaf chlorophyll content (*ACC_m_*) of offspring ramets (F_2_ generation) experiencing parental light environment significantly increased than those of offspring ramets (F_2_ generation) experiencing non-parental light environment (Figures 3A,B). Opposite pattern was observed in area-based leaf chlorophyll content (*ACC_a_*) and mass-based leaf chlorophyll content (*ACC_m_*) of offspring ramets (F_2_ generation) from parental ramets subjected to high light treatment (Figures 3A,B).

Leaf nitrogen content per unit area (*N_A_*) of offspring ramets (F_2_ generation) was significantly affected by light treatment experienced by F_0_ generation, light treatment experienced by F_2_ generation and their interaction (F_0_ × F_2_) (Table 2). However, leaf nitrogen content per unit mass (*N_M_*) of offspring ramets (F_2_ generation) was significantly affected by light treatment experienced by F_2_ generation (F_2_) (Table 2). When parental ramets (F_0_ generation) were subjected to low light treatment, leaf nitrogen content per unit area (*N_A_*) of offspring ramets (F_2_ generation) experiencing parental light environment was significantly greater than that of offspring ramets (F_2_ generation) experiencing non-parental light environment (Figure 3D). Leaf nitrogen content per unit mass (*N_M_*) of offspring ramets (F_2_ generation) subjected to low light treatment was significantly greater than that of offspring ramets subjected to high light treatment (Figure 3C).

### Photosynthetic Property

**FIGURE 4:**
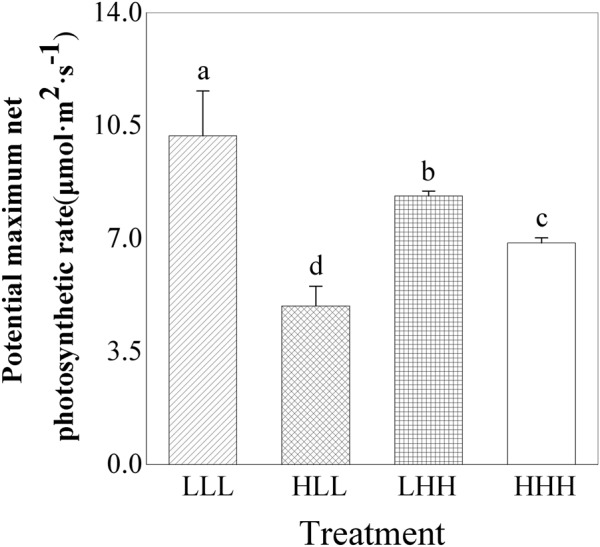
Effects of transgenerational plasticity on potential maximum net photosynthetic rate of offspring ramets (F_2_ generations). The same lower case letters are not significantly different at the *P* = 0.05 level. Values are means ± s.e. (standard errors), *n* = 8. HHH: F_0_ generation high light+ F_1_ generation high light+ F_2_ generation high light; HLL: F_0_ generation high light+ F_1_ generation low light+ F_2_ generation low light; LHH: F_0_ generation low light+ F_1_ generation high light+ F_2_ generation high light; LLL: F_0_ generation low light+ F_1_ generation low light+ F_2_ generation low light.

Potential maximum net photosynthetic rate (*P_max_*) of offspring ramets (F_2_ generation) was significantly affected by light treatment experienced by F_0_ generation and interaction between light treatment experienced by F_0_ generation and light treatment experienced by F_2_ generation (F_0_ × F_2_) (Table 2). Potential maximum net photosynthetic rate (*P_max_*) of offspring ramets (F_2_ generation) from parental ramets subjected to low light treatment was greater than that of offspring ramets (F_2_ generation) from parental ramets subjected to high light treatment (Figure 4). Potential maximum net photosynthetic rate (*P_max_*) of offspring ramets (F_2_ generation) subjected to parental light environment was greater than that of offspring ramets subjected to non-parental light environment (Figure 4).

### Growth Performance

**FIGURE 5:**
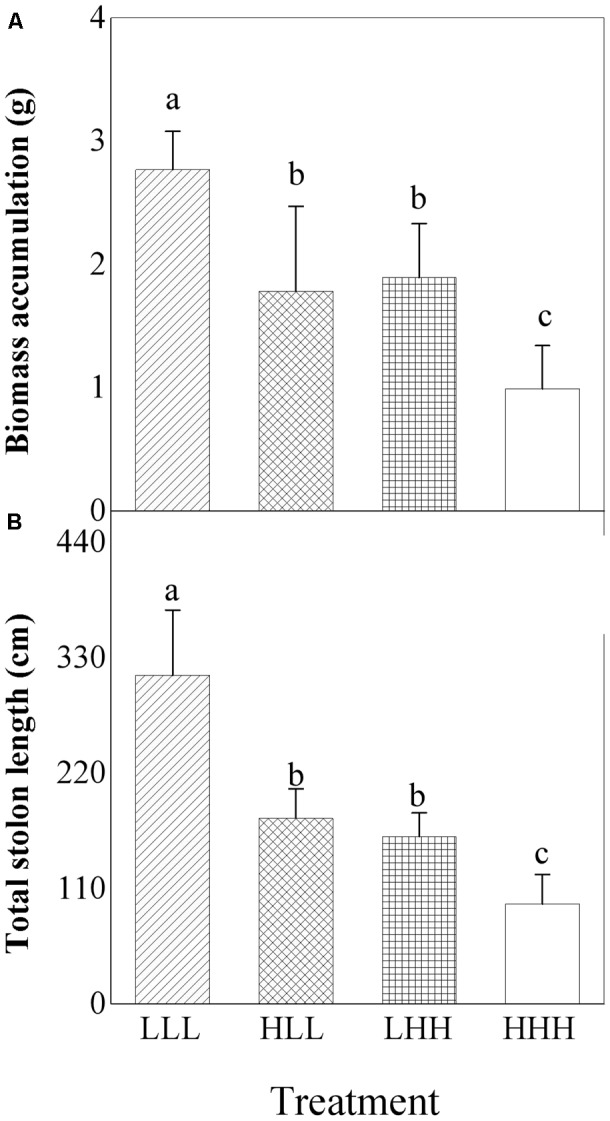
Effects of transgenerational plasticity on growth performance of offspring ramets (F_2_ generation). The same lower case letters are not significantly different at the *P* = 0.05 level. Values are means ± s.e. (standard errors), *n* = 8. HHH: F_0_ generation high light+ F_1_ generation high light+ F_2_ generation high light; HLL: F_0_ generation high light+ F_1_ generation low light+ F_2_ generation low light; LHH: F_0_ generation low light+ F_1_ generation high light+ F_2_ generation high light; LLL: F_0_ generation low light+ F_1_ generation low light+ F_2_ generation low light.

Biomass accumulation and total stolon length of offspring ramets were significantly affected by light treatment experienced by F_0_ generation (F_0_) and light treatment experienced by F_2_ generation (F_2_) (Table 2). The greatest biomass accumulation and total stolon length were observed in offspring ramets (F_2_ generation) subjected to low light treatment as parental ramets (F_0_ generation) experienced (Figures 5A,B). When parental ramets (F_0_ generation) were subjected to low light treatment, biomass accumulation and total length of stolon of offspring ramets (F_2_ generation) experiencing parental light environment were significantly greater than those of offspring ramets (F_2_ generation) experiencing non-parental light environment (Figures 5A,B). Opposite pattern was observed in offspring ramets (F_2_ generation) from parental ramets subjected to high light treatment (Figures 5A,B).

## Discussion

For stoloniferous herb *C.asiatica*, the experiment demonstrated transgenerational plasticity triggered by high/low light treatment. Light environment experienced by parental ramets (F_0_ generation) significantly influenced morphological and physiological properties of offspring ramets (F_2_ generation) as well as growth performance even after they were detached from the parental ramets. The results supported our first hypothesis that effects of transgenerational plasticity on morphological and physiological properties can transmit across vegetative generations of *C. asiatica*. Due to limited opportunities to adapt to environmental changes genetically, transgenerational plasticity can impose substantial impact on population dynamics (Benton et al., 2005; Plaistow et al., 2006) and evolution of clonal plants in response to environmental changes (Wade, 1998; Räsänen and Kruuk, 2007). So, transgenerational plasticity would have consequences for population dynamics, and ultimately, evolution, especially given the limited levels of genotypic variation in clonal plants (González et al., 2017).

When parental ramets (F_0_ generation) were subjected to low light treatment, offspring ramets (F_2_ generation) experiencing parental light environment presented better growth performance than offspring ramets (F_2_ generation) experiencing non-parental light environment. This is consistent with previous study that if offspring ramets spread into a new environment as experienced by parental ramets, transgenerational plasticity may facilitate establishment of their populations by enabling adaptation to the new environment more rapidly than natural selection (Latzel and Klimešová, 2010). In addition, opposite pattern was observed in offspring ramets (F_2_ generation) from parental ramets subjected to high light treatment. The results supported our second hypothesis that effects of transgenerational plasticity on growth performance are context-dependent. Such transgenerational plasticity spanning across vegetative generations is likely adaptive in clonal species (Herman and Sultan, 2011; Holeski et al., 2012).

Compared to offspring ramets (F_2_ generation) from parental ramets (F_0_ generation) subjected to high light treatment, growth performance of offspring ramets (F_2_ generation) from parental ramets (F_0_ generation) subjected to low light treatment was favored in parental or non-parental light environment. The results supported our third hypothesis. Habitat-specific DNA methylation of clonal genotypes from natural populations may result in locally specialized ecotypes (Verhoeven and Preite, 2014). Further, transgenerational plasticity can affect the evolutionary rate and direction of clonal plants (Latzel et al., 2016; González et al., 2017).

Clonal species often adopted morphological response such as stolon elongation or petiole expansion to escape from environmental stress such as flooding (Luo et al., 2009), low light (González et al., 2017), metal pollutions (Roiloa and Retuerto, 2012) and interspecific competition from neighbor species (Evans and Cain, 1995). Seedlings from parents grown in a CO_2_-elevated environment reduced photosynthesis compared to seedlings from parents grown in ambient CO_2_ conditions (Huxman et al., 2001). Parents may enable their offspring to adapt to environmental changes through morphological and photosynthetic adjustment. With plastic changes of morphological properties, photosynthetic capacity and growth performance were significantly improved in offspring ramtes (F_2_ generation) experiencing parental light environment than in offspring ramtes (F_2_ generation) experiencing non-parental light environment when parental ramets (F_0_ generation) were subjected to low light treatment. We tentatively concluded that in response to low light treatment, variation of morphological and physiological properties in parental ramets was transmited to their offspring ramets (Latzel et al., 2009). When parental ramets (F_0_ generation) were subjected to low light treatment, the greatest leaf nitrogen content per unit area (*N_A_*) was observed in offspring ramtes (F_2_ generation) experiencing parental light environment with a decrease of specific leaf area (SLA). The results implied that effects of transgenerational plasticity on photosynthesis of offspring ramets might be mediated by alternation of resources allocation toward the photosynthetic apparatus (Latzel et al., 2009). It is suggested that environmentally induced epigenetic change and/or inherited resource allocation pattern toward photosynthesis may be responsible for effects of transgenerational plasticity on photosynthesis of offspring ramets.

Furthermore, clonal plants have the potential to selectively place ramets and to avoid unfavorable conditions through morphological plasticity of spacer or branching intensity (Hutchings and Kroon, 1994; Kleunen and Fischer, 2001). For *Polygonum persicaria*, progeny from parent experiencing drought environment produced longer, more rapidly extending root systems and greater biomass in parental environment than those of progeny in non-parental environment (Galloway and Etterson, 2007). In addition, root-shoot biomass ratio and specific root length of progeny subjected to drought environment experienced by parent significantly increased than those of progeny in ample water environment (Sultan et al., 2009). In our experiment, effects of transgenerational plasticity on morphological and physiological properties of offspring ramets (F_2_ generation) depended on environmental characteristics experienced by their parent and themselves. Clonal plants thus have the potential to reflect past and current environmental conditions even anticipate future conditions (Latzel et al., 2016). So, the dynamics and genetics of clonal populations may be affected by the interaction of genotypes to phenotypes.

To the best of our knowledge, there has been rare study directly examining the effects of transgenerational plasticity on clonal plants through both morphological and physiological properties. Our work provides evidence that transgenerational plasticity through both morphological and physiological flexibility was triggered across vegetative generations for stoloniferous herb *C. asiatica* subjected to high/low light treatment. Life-history traits such as clonal integration, intraclonal division of labor and clonal architecture et al may be advantageous to exploitation and colonization of clonal plants in heterogeneous habitats (Latzel and Klimešová, 2010; Martina and Ende, 2013; Chen et al., 2015). The transgenerational plasticity can allow offspring ramets to present adaptive phenotype early without lag time in response to the current environment. Thus, it is very important for clonal plants in adapting temporally and spatially heterogeneous habitats. A wider range of species are needed to understand the generality of this pattern and to assess fully the ecological advantages afforded by these features.

## Author Contributions

All authors conceived, designed, and performed the experiments and wrote the paper.

## Conflict of Interest Statement

The authors declare that the research was conducted in the absence of any commercial or financial relationships that could be construed as a potential conflict of interest.
